# Bearing Fault Diagnosis with a Feature Fusion Method Based on an Ensemble Convolutional Neural Network and Deep Neural Network

**DOI:** 10.3390/s19092034

**Published:** 2019-04-30

**Authors:** Hongmei Li, Jinying Huang, Shuwei Ji

**Affiliations:** 1 School of Computer and Engineering Control, North University of China, Taiyuan 030051, China; hongmeili@tyust.edu.cn (H.L.); lhmxiawa@163.com (S.J.); 2 School of Mechanical Engineering, North University of China, Taiyuan 030051, China

**Keywords:** bearing fault diagnosis, convolutional neural network, deep neural network, feature fusion, dynamic ensemble

## Abstract

Rolling bearings are the core components of rotating machinery. Their health directly affects the performance, stability and life of rotating machinery. To prevent possible damage, it is necessary to detect the condition of rolling bearings for fault diagnosis. With the rapid development of intelligent fault diagnosis technology, various deep learning methods have been applied in fault diagnosis in recent years. Convolution neural networks (CNN) have shown high performance in feature extraction. However, the pooling operation of CNN can lead to the loss of much valuable information and the relationship between the whole and the part may be ignored. In this study, we proposed CNNEPDNN, a novel bearing fault diagnosis model based on ensemble deep neural network (DNN) and CNN. We firstly trained CNNEPDNN model. Each of its local networks was trained with different training datasets. The CNN used vibration sensor signals as the input, whereas the DNN used nine time-domain statistical features from bearing vibration sensor signals as the input. Each local network of CNNEPDNN extracted different features from its own trained dataset, thus we fused features with different discrimination for fault recognition. CNNEPDNN was tested under 10 fault conditions based on the bearing data from Bearing Data Center of Case Western Reserve University (CWRU). To evaluate the proposed model, four aspects were analyzed: convergence speed of training loss function, test accuracy, F-Score and the feature clustering result by t-distributed stochastic neighbor embedding (t-SNE) visualization. The training loss function of the proposed model converged more quickly than the local models under different loads. The test accuracy of the proposed model is better than that of CNN, DNN and BPNN. The F-Score value of the model is higher than that of CNN model, and the feature clustering effect of the proposed model was better than that of CNN.

## 1. Introduction

Rolling bearings have been widely applied in various rotating devices, which are used to support the rotating bodies and transmit torque and power in transmission systems [1,2]. A bearing failure can lead to unnecessary downtime, serious economic losses and even casualties [3]. Therefore, reliable bearing condition monitoring is required.

Recently, deep learning has been widely applied in pattern recognition [4,5,6]. Deep learning is a new field of machine learning. It is a multi-level feature learning method which uses simple but non-linear components to transform the features of each layer (from the original data) into more abstract higher-order hierarchical features [7]. Therefore, deep learning has a good feature learning ability. DNN, deep belief network (DBN), CNN, and deep auto-encoder are the main models of deep learning.

Among various deep-learning models, CNN [8] originally used in image recognition has been successfully applied in extracting feature. Their unique modeling characteristics can help to discover local structures or configurable relations in observations, thus CNN is now the main model in image analysis, video analysis, and speech recognition. CNN-based fault diagnosis methods have been investigated in recent years. Chen et al. [9] used CNN to identify and classify gearbox faults. Firstly, statistical measurements in time domain and frequency domain were extracted manually from vibration signals as CNN’s input. Then, CNN learned and extracted features automatically from these statistical measurements. Janssens et al. [10] used CNN model in bearing fault detection with vibration signals. CNN model worked on the frequency spectrum obtained from vibration data by Discrete Fourier Transform. Zhang et al. [11] converted the vibration signal into an image and used it as a CNN input for bearing fault diagnosis. Han et al. [12] proposed a dynamic ensemble convolutional neural network (DECNN) model based on CNN and wavelet transform to identify gearbox faults under variable speed. The DECNN model consists of several parallel CNNs and the model input is a multi-level wavelet coefficient matrix constructed by wavelet packet transform. To solve the non-stationary characteristics, Xie et al. [13] studied the feature extraction method of bearing based on empirical mode decomposition (EMD) and CNN. The effective intrinsic mode functions obtained by EMD are selected and reconstructed and the spatial information is extracted from frequency spectrum by CNN. Then, the features extracted from both methods are combined together to realize non-stationary signal feature extraction and fault diagnosis. Xia et al. [14] combined the rolling bearing vibration signals collected by multiple sensors as the input of CNN to achieve the higher and more robust diagnostic performance. Guo et al. [15] studied and improved the CNN structure and proposed a new hierarchical learning rate adaptive deep convolutional neural network, which can not only diagnose bearing failure but also determine its severity. Based on the different signal characteristics of bearing, Wang et al. [16] used particle swarm optimization algorithm to determine the main parameters of the CNN model. In the above studies, a two-dimensional convolution structure is used in image processing, thus the two-dimensional convolution structure is selected for mechanical fault diagnosis. One-dimensional (1D) CNN has been successfully applied in the classification of bearing fault detection since most of the measured data of mechanical faults are time-varying one-dimensional parameters. Turker et al. [17], Levent et al. [18] and Jing et al. [19] successfully used 1DCNN in the classification of bearing failure detection.

Although CNN have made great achievements in fault diagnosis, CNN pays more attention to local features [20,21,22]. When data dimension is reduced, the pooling layer of CNN may lose a lot of valuable information and ignore the relationship between the whole signal and a part of the signal. For the same kind of failures with different degrees of severity, target descriptions based on details are ambiguous, thus affecting the accuracy of fault diagnosis. In previous studies on mechanical fault diagnosis based on CNN, mechanical vibration signals were converted into two-dimensional matrices or images, thus increasing the work load and leading to the wrong expression of information.

Time domain statistical features can reflect the signal amplitude fluctuation, impact intervals and energy distribution law, and have been approved as simple and effective features for fault diagnosis [23,24,25]. For example, square root amplitude value and absolute mean amplitude value can measure the vibration amplitudes and energy of time domain signals. Peak-to-peak amplitude is the distance from the top of the positive peak to the bottom of the negative peak. Kurtosis reflects the degree to which the signal deviates from the normal distribution. Skewness and shape factor indicate the degree to which the center of the signal probability density function deviates from the normal distribution. With DNN, global features can be efficiently extracted from time-domain statistical features of signals.

Since CNN shows defects in fault diagnosis, we attempted to integrate different deep learning models to improve the prediction accuracy. It is reported that integrating various models can increase the prediction accuracy [26,27,28,29]. We proposed a CNNPEDNN model for DNN parallel ensemble CNN based on feature fusion. In CNNEPDNN model, a fusion layer is added to integrate DNN with CNN and the global features extracted by DNN from time-domain statistical features are combined with the local features extracted by CNN from vibration signals. These abstract features can further enhance the identification ability among different fault states. The proposed model was verified with the bearing data of Case Western Reserve University (CWRU) under different load conditions and compared against CNN.

The rest of this paper is organized as follows. Section 2 elaborates the basic knowledge of DNN and CNN. Section 3 presents the proposed CNNEPDNN model with detailed description. Section 4 describes the experimental setup and time-domain statistical features and presents the evaluation results on four sets of experiments. The advantages of the CNNEPDNN model were demonstrated. Finally, the conclusions are drawn in Section 5.

## 2. Fundamental Theories

### 2.1. DNN Model

**Figure 1:**
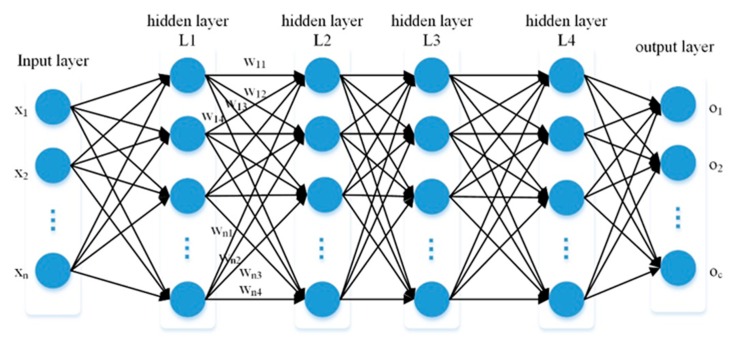
Structure diagram of DNN.

Similar to the shallow neural network layer, the neural network layer inside DNN is divided into three categories: input layer, hidden layer and output layer. DNN has a deep structure composed of a number of hidden layers. It is generally believed that a deep network contains at least three hidden layers, whereas a very deep network should contain at least 10 hidden layers [30]. Through multiple hidden layers, DNN can learn more complex functional relations. Goodfellow et al. [31] indicated that, in certain problems, the more hidden layers of the network there were, the higher the accuracy was. DNN structure is shown in Figure 1. The number of neurons in the input layer is determined by the characteristics of sample data. Each hidden layer contains multiple neurons and the number of neurons can be obtained from an empirical formula [31]. The output of each hidden layer is nonlinear transformed through an activation function and common nonlinear activation functions include sigmoid, Rectified Linear Unit (ReLU), etc. The number of neurons in the output layer is determined by the number of sample labels. The output layer and the last hidden layer are connected to logistic regression.

$x_{1},x_{2},\ldots ,x_{n}$ and $o_{1},o_{2},\ldots ,o_{c}$ represent the input and output of the network, respectively. The feature extraction operation of DNN is expressed as:

(1) $$f(x)=\varphi (w_{ij}^{L}x+b^{L})$$

where $w_{ij}$ is the connection weight between the L−1 hidden layer neural cell i and the L hidden layer neural cell j; $b^{L}$ is the bias of L hidden layer neurons; φ is denoted as activation function; and f(x) is the output of the L hidden layer neural cell j.

### 2.2. CNN

**Figure 2:**
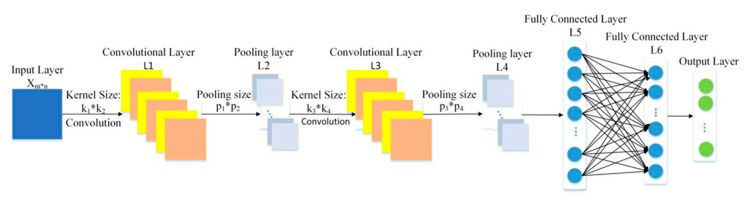
A typical architecture of CNN.

CNN has two network layers with a special structure, namely convolution layer and pooling layer. The convolution layer is so named because it uses convolution operation instead of matrix multiplication. Convolution layer and pooling layer are the core modules for realizing the CNN feature extraction function. In general, alternating connection means that a convolution layer is connected to a pooled layer and a pooled layer is then connected to a convolutional layer. Both convolutional layer and pooling layer are composed of multiple two-dimensional planes and each feature map is a plane. The numbers of convolutional layer and pooling layer can be determined according to actual demands. Generally, CNN is composed of input layer, convolution layer, pooling layer, fully connected layer and output layer. A typical CNN model is illustrated in Figure 2.

Each convolution layer contains multiple convolution kernels, which are weight matrices. Different convolution kernels have different weights. The convolutional layer extracts features through the convolution kernel, which slides on the feature map of the previous layer and performs convolution operation on the local region corresponding to the feature map. After the sliding is completed, the convolution transformation is carried out on the feature map from the previous layer and then the convolution result is nonlinearly changed to obtain the feature map of the convolution layer. Different convolution kernels correspond to different feature maps. A convolution layer has the characteristics of weight sharing and local connection and the convolution operation is defined as:

(2) $$x_{k^{\prime }}^{L}=\varphi (\sum_{k\in M_{j}}w_{kk^{\prime }}*x_{k}^{L-1}+b_{k^{\prime }}^{L})$$

where $x_{k}^{L-1}$ is defined as the output of the k feature map at the L−1 layer; w is defined as the convolution kernel; $w_{kk^{\prime }}^{l}$ is defined as the kernel from the $k^{\prime }$ feature map at L layer to the k feature map at L−1 layer; ∗ is defined as the convolution operation; $b_{k^{\prime }}^{L}$ is defined as the bias of the $k^{\prime }$ at L layer; φ is defined as the nonlinear activation function; $x_{k^{\prime }}^{L}$ is defined as the $k^{\prime }$ feature map at L layer; and $M_{j}$ is defined as the number of input feature maps.

The pooling layer is introduced to reduce the dimension of the feature map representation. In the pooling operation, a matrix window is used to scan the feature map and then a statistic is selected from the rectangular region as the output of the rectangular region to reduce the number of elements. The pooling operation is defined as:

(3) $$x_{k}^{L}=\phi (x_{k}^{L-1})$$

where $x_{k}^{L-1}$ is the k feature map at L−1 layer; ϕ is pooling operation; and $x_{k}^{L}$ is the k feature map at L layer. The pooling operations generally include maximum pooling and mean pooling. Maximum pooling looks for the maximum value in each matrix window and average pooling is to take the average value of each matrix window. Pooling operations are invariant under small shifts and distortions and can avoid overfitting.

Convolution layers and pooling layer are often followed by several fully connected layers. The fully connected layers usually transform the output of two-dimensional feature map of convolution layer or pooling layer into one-dimensional vectors. All neurons of the fully connected layer are fully connected to neurons in the previous and subsequent layers, which can be regarded as the hidden layer in the DNN.

### 2.3. Forward Transmission Process and Back Propagation of CNN and DNN

In this study, the training methods of CNN and DNN are supervised training methods, which require training samples (i.e., known data and their corresponding labels) to obtain an optimal model. The forward transmission processes of CNN and DNN are to input samples into the network, process them through each network layers, and finally obtain the output. The output layer and the last hidden layer are connected through Softmax logical regression. In a C-class classification problem, as for the training set $D={\left\{X,Y\right\}}^{N}$, where N is the number of the training sample; $X\in {\mathbb{R}}^{N\times 1\times L}$ is the input data; $Y\in {\mathbb{R}}^{N\times 1}$ is the health condition label of the X; and the forward transmission processes of CNN and DNN are denoted as:

(4) $$f(x)=f_{L-1}(f_{L-2}(\ldots f_{1}(x,\theta_{1}^{t}),\theta_{L-2}^{t}),\theta_{L-1}^{t})$$

(5) $$O^{L}=soft\max(f(x,\theta_{L}^{t}))=\frac{\exp f_{L}(x,\theta_{L}^{t},c)}{\sum_{j=1}^{C}\exp f_{L}(x,\theta_{L}^{t},j)}$$

where $\theta_{1}^{t},\theta_{2}^{t},\ldots ,\theta_{L-1}^{t},\theta_{L}^{t}$ are defined as the learnable parameters of each L network layer in the training t stage, such as weight w and biases b; $f_{1},f_{2},\ldots ,f_{L-1},f_{L}$ are operations at each network layer, such as convolution operation and pooling operation of CNN and dot product operation of DNN; x is the sample data provided by the input layer; $f_{L}(x,\theta_{L}^{t})$ represents the output of L network layer with parameters on input x; and $O^{L}$ is the classification result of the output layer.

CNN and DNN fine-tune network parameters based on the loss function between the minimized network output and the expected output and cross entropy loss is widely used as the loss function of network. The error between the network output and the expected output is distributed to each layer by backpropagation on m batches of the dataset D. CNN and DNN optimization problems are expressed as:

(6) $$O^{L}=soft\max(f(x,\theta_{L}^{t}))=\frac{\exp f_{L}(x,\theta_{L}^{t},c)}{\sum_{j=1}^{C}\exp f_{L}(x,\theta_{L}^{t},j)}$$

The CNN and DNN continue to perform the processes of forward propagation and back propagation until the loss function converges or reaches the specified iterative termination condition, thus realizing the network supervision training.

## 3. CNNEPDNN Model

**Figure 3:**
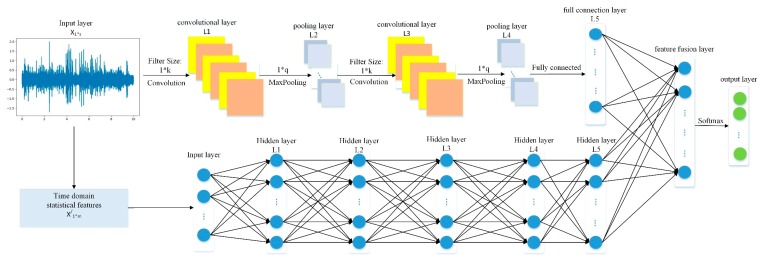
Schematic diagram of the proposed model CNNEPDNN.

The architecture of the CNNEPDNN model is shown in Figure 3. DNN is connected with CNN through a fusion layer to construct a global model. The CNN consists of an input layer, two convolutional layers and two pooling layers and adopts 1D convolution structure with the vibration signal as the input. The DNN consists of an input layer and multiple hidden layers with the time domain statistical features of the vibration signal as the input. Then, the fusion layer is used to connect the two local networks together for feature fusion and Softmax logical regression is used for classification. To avoid overfitting, dropout [32] is used in the fusion layer. The detailed parameters of the network structure of model CNNEPDNN are shown in Table 1.

**Figure 4:**
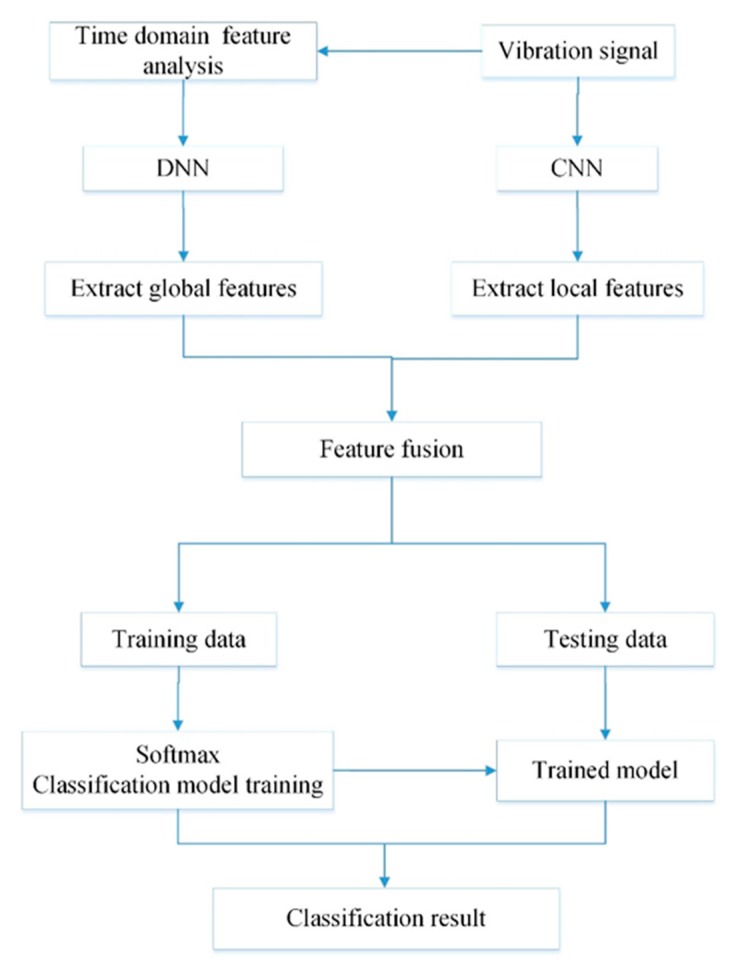
Fault diagnosis process of CNNEPDNN model.

The CNNEPDNN model also iteratively implements forward propagation and back propagation, similar to other training methods of DNN and CNN. The fault diagnosis process of CNNEPDNN model is shown in Figure 4. The forward propagation of CNNEPDNN local network is the same as that of a single network model. It processes and extracts features successively from the input layer to the hidden layer, and then integrates the features extracted from the two local networks through a fully connected layer. Assuming that a training set $\bar{D}=\{X,X^{\prime },Y\}$ has N samples, where N represents the training sample of vibration sensor signal; $X\in {\mathbb{R}}^{N\times 1\times K}$ represents the time-domain statistical feature training sample extracted from the vibration sensor signal X; and $Y\in {\mathbb{R}}^{N\times 1}$ represents the training sample labels. At iteration t, the forward propagation process of CNNEPDNN model can be defined as follows:

(7) $$f(x,x^{\prime })=f_{L-1}(f_{c,L-2}(\ldots f_{c,1}(x,\theta_{c,1}^{t}),\theta_{c,L-2}^{t}),f_{d,L-2}(\ldots f_{d,1}(x^{\prime },\theta_{d,1}^{t}),\theta_{d,L-2}^{t}),\theta_{L-1}^{t})$$

(8) $$O^{L}=\frac{\exp f_{L}(x,x^{\prime },\theta_{L}^{t},c)}{\sum_{j=1}^{C}\exp f_{L}(x,x^{\prime },\theta_{L}^{t},j)}$$

where $\theta_{c,1}^{t},\theta_{c,2}^{t},\ldots ,\theta_{c,L-1}^{t}$, $\theta_{d,1}^{t},\theta_{d,2}^{t},\ldots ,\theta_{d,L-1}^{t}$, and, respectively, represent the learnable parameters of the local networks and the fusion layer in the CNNEPDNN model; $f_{c,L-1}$ and $f_{d}^{L-1}$ are the operations at each network layer of CNN and DNN; x and, respectively, represent the input samples of CNN and DNN; $f_{L}(x,x^{\prime },\theta_{L}^{t})$ represents the output of L network layer with parameters on input x and $x^{\prime }$; and $O^{L}$ is the classification result of the output layer.

For convenience, all network parameters of CNNEPDNN are defined as $\bar{\theta }$ and the loss of the CNNEPDNN model $f(\bar{\theta })$ on the data $\bar{D}$ is denoted as $L(f(x,x^{\prime },\bar{\theta }),y)$. For the feature fusion, the global model sends the loss back to the local worker through the fusion layer, and then the parameters of the local models are broadcasted to each local network on m batches of the dataset $\bar{D}$.

(9) $$L(f(x,x^{\prime },\bar{\theta }),y)=-\frac{1}{m}\sum_{i=1}^{m}\sum_{c=1}^{c}y_{c}\ln f(x,x^{\prime },\bar{\theta },c)$$

## 4. Fault Diagnosis Based on CNNEPDNN

To verify the CNNEPDNN model in fault diagnosis, the proposed model was used to diagnose the health of rolling bearings. The experimental setup and process are described in the following sections.

### 4.1. Experimental Setup

**Figure 5:**
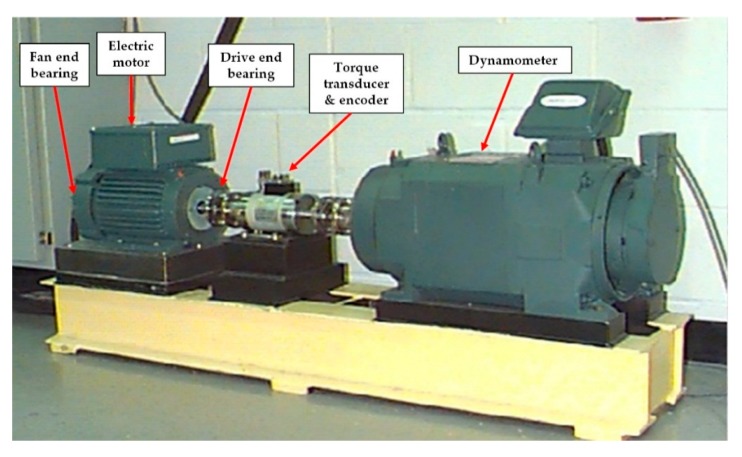
Experimental platform for acquiring vibration signals from rolling bearings.

**Figure 6:**
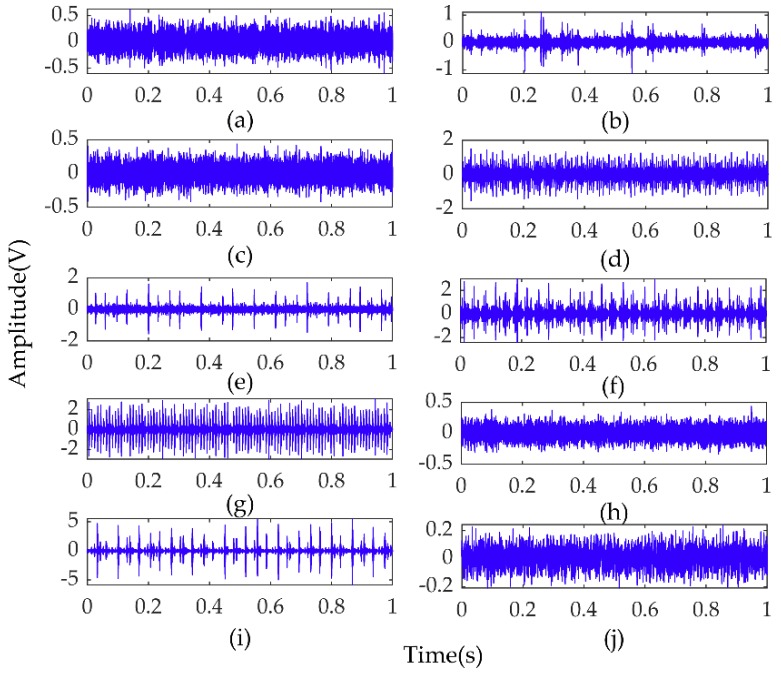
Vibration signals of bearing under 2-hp loads from one sensor. (**a**–**c**) are the bearing inner race fault signal under fault size of 7mils, 14mils and 21 mils, respectively. (**b**–**d**) are the bearing outer race fault under fault size of 7 mils, the 14 mils and 21 mils, respectively. (**g**–**i**) are the bearing ball fault under size of 7 mils, 14 mils and 21 mils, respectively. (**j**) the normal bearing signal.

The experiment was carried out with the rolling bearing data collected by the Bearing Data Center of CWRU [33]. As shown in Figure 5, the test platform was composed of 2-hp (1.5 kw) motor (1797–1722 rpm), torque sensor, accelerometer sensor, power tester, etc. The motor shaft was supported by 6205-2rs JEM SKF type bearings. In the experiment, the acceleration sensors were installed at 12 o’clock position above the motor drive end (DE) and fan end (FE) through a magnetic base. Motor bearings were artificially seeded with a single point fault, respectively, on the outer race (OR), the inner race (IR), and the ball by electric discharge machining (EDM). The fault diameters were 7, 14, and 21 mil and the depth was 11 mil. Vibration signals under four motor loads (0, 1, 2 and 3 hp) were collected with a 16-channel DAT recorder and the sampling frequencies were 12 kHz. The vibration signals of ten conditions under 2-hp load from one sensor are shown in Figure 6.

Vibration signal datasets collected under four loads (3, 2, 1 and 0 hp) are represented by A, B, C and D, respectively. Under each load, the fault conditions included normal, the inner race fault, the outer race fault and the ball fault, wherein the inner race, the outer race and the ball faults were further categorized by the fault size (7, 14, and 21 mils). Therefore, we had ten fault conditions for each load. For each pattern and load configuration, the collected signals were divided into segments; 512 points were selected as a segment and one segment as a sample. There were 237 samples for each condition and 2370 samples in total for ten health conditions under one load. The specific experimental data are shown in Table 2. Next, Time domain statistical features of each sample were calculated. Generally, according to dimensional and non-dimensional features, time domain statistical features were divided into two parts. Dimensional statistical parameters include maximum, minimum, peak-to-peak, mean, mean square and variance. Non-dimensional statistical parameters include waveform indicators, peak indicators, pulse indicators, margin indicators, kurtosis indicators, and skewness indicator. The selected nine time-domain statistical features of each sample were calculated according to the formulas in Table 3. In the experiments, we randomly selected 2000 samples from 2370 original vibration signals and time domain feature samples as training sets and the remaining samples as test sets to validate the proposed model under four motor loads. To reduce the impact of randomness, 10 experiments were conducted on each dataset.

### 4.2. Diagnostic Results and Analysis

The proposed model was compared with CNN model in four aspects: convergence speed of training loss function, test accuracy, F-Score and feature learning ability. The simulations were implemented in 64-bit PyCharm with a computer with I7-8550U at 1.8 GHZ (4 cores) and 8-Gb memory.

#### 4.2.1. Convergence Speed of Training Loss Function

**Figure 7:**
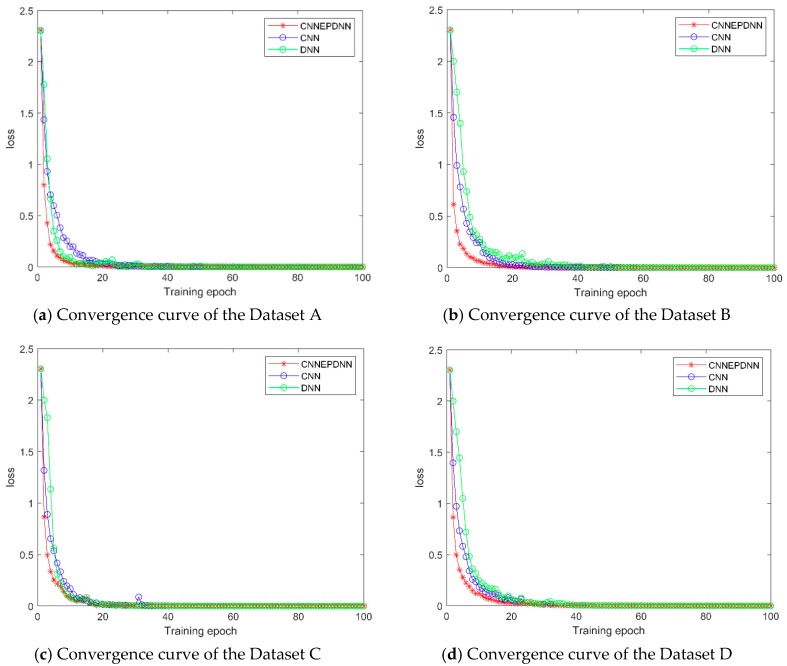
Comparison of loss function between CNN, DNN and CNNEPDNN. (**a**), (**b**), (**c**), (**d**) are the convergence curve of training loss function on dataset A, B, C and D, respectively.

The convergence curve of the training process in a certain experiment was randomly selected to analyze the convergence rate. As shown in Figure 7, the convergence of CNNEPDNN model was achieved within 20 iterations and it was faster than CNN and DNN under different loads. In addition, the model is a parallel structure and the cross-entropy loss is convex, which ensured that the performance of the global model was better than that of the local models, and had no effect on the computational complexity [34]. Experimental results confirm that the one time of CNNEPDNN training (one forward propagation and one back propagation) was basically the same as the CNN network structure with an average time of 7–12 ms; the average training time of DNN was 3 ms.

#### 4.2.2. Test Accuracy

**Figure 8:**
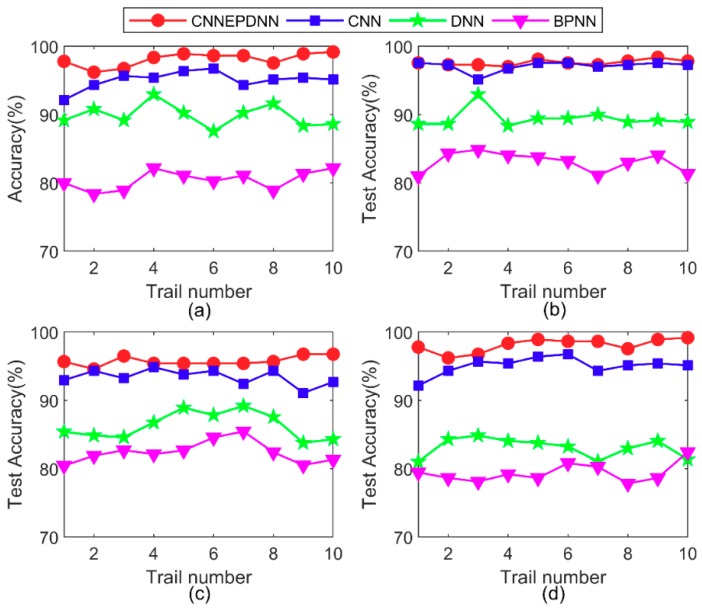
Testing accuracy results of the proposed method and CNN, DNN, BPNN in 10 trials: (**a**), (**b**), (**c**) and (**d**) are the test accuracy of four methods on dataset A, B, C and D, respectively.

To test the effectiveness and superiority of CNNEPDNN, CNN, DNN, and BPNN were selected to compare with the proposed model. Figure 8 presents the testing results of the ten trails of all comparative methods on four datasets. The average test accuracy and standard deviation of all comparison methods in the experiment are shown in Table 4. The results show that the proposed method could improve the accuracy and reliability of diagnosis results.

#### 4.2.3. F-Score

In addition to accuracy analysis, two other useful indexes are precision and recall. On the one hand, it is not desirable to have too many false alarms (high recall rate, low precision) because this will increase the operating cost due to unnecessary downtime. On the other hand, if only real faults are marked and no false positive results are reported, the accuracy is high, but the recall rate is low. It takes much time to balance these two indicators comprehensively. F-Score [10] comprehensively considers the harmonic values of precision and recall so that the alarm will not be triggered until an actual fault occurs without any missing fault or false alarm. Precision, Recall and F-Score are defined as follows:

(10) $$\mathrm{Pr}ecision=\frac{\left|TP\right|}{\left|TP\right|+\left|FP\right|}$$

(11) Recall=|TP||TP|+|FN|

(12) F−Score=(1+β2)Precision×RecallPrecision+Recall

where |TP| is the true positive classification; |TN| is the number of true negative classifications; |FP| is the number of false positive classifications, such as false positive classification; and |FN| is the number of false negative classifications, such as missed faults. When β=1, F-Score combines precision and recall values so that the alarm will not be triggered until an actual fault occurs without any missing fault or false alarm. As shown in Table 5, precision, recall and F-Score of CNNEPDNN model are higher than those of CNN model under different loads.

**Figure 9:**
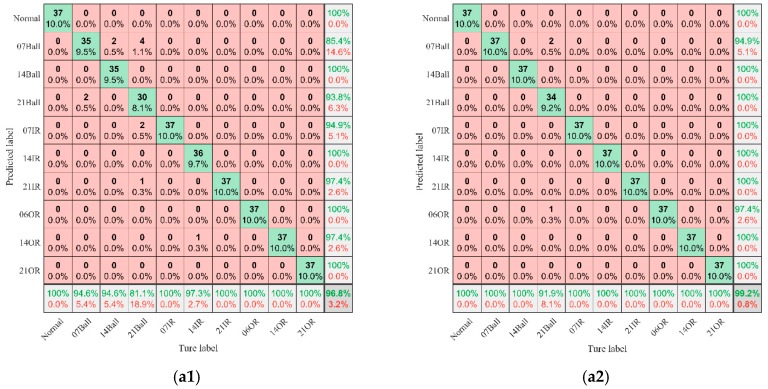

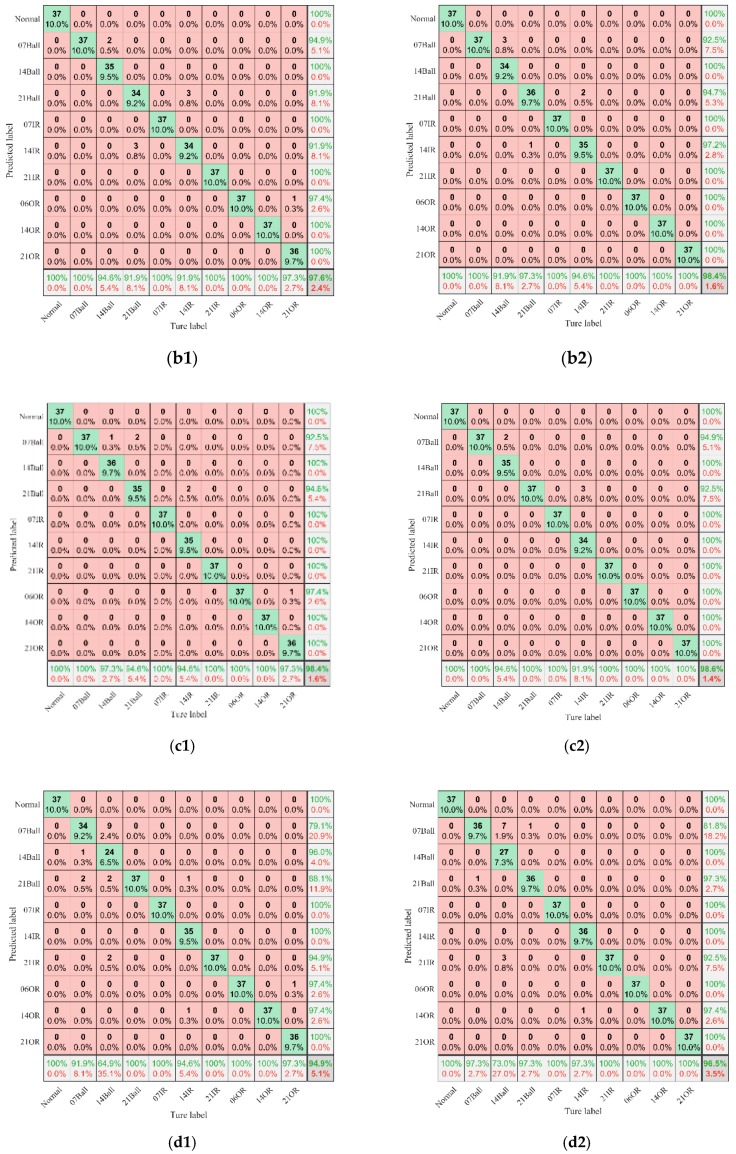
Confusion matrix of the bearing fault classification with CNN and CNNEPDNN. (**a1**–**d1**) are the confusion matrix using CNN on dataset A,B,C and D, respectively. (**a2**–**d2**) are the confusion matrix of CNNEPDNN on dataset A,B,C and D, respectively.

To further evaluate the proposed model, the confusion matrices of the test dataset for one trial are shown in Figure 9. Each column of the confusion matrix represents the prediction category and each row represents the real category to which the data belongs. The green data in the last row indicates the precision of each fault state and the green data in the last column indicates the recall of each fault. We can see the diagnosis results of each condition from the confusion matrix. Figure 9(a1–d1) shows the confusion matrix of CNN for fault identification of Datasets A–D and Figure 9(a2–d2) shows the confusion result of CNNEPDNN for fault identification of Datasets A–D.

**Figure 10:**
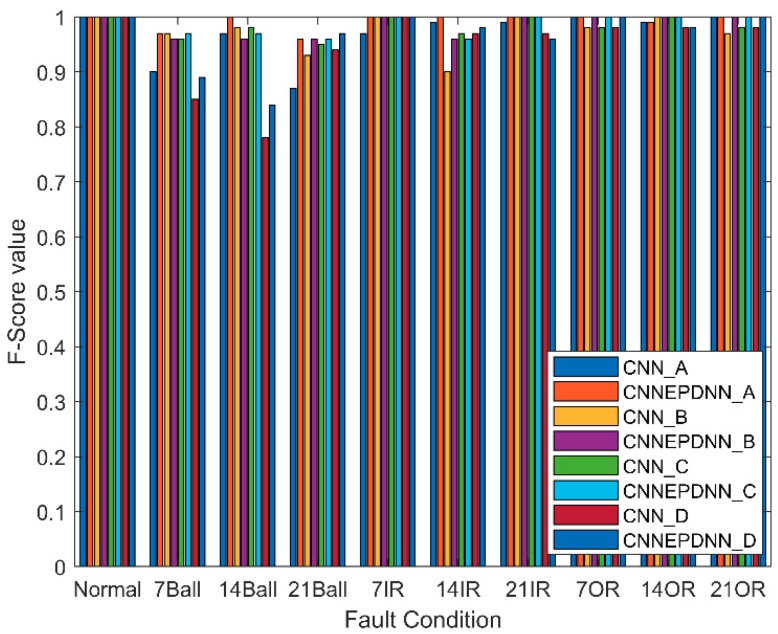
F-Score value of ten condition fault in four dataset.

F-score can be calculated according to precision and recall of each fault condition, F-score value for ten fault condition in four datasets is shown in Figure 10. The F-Score values of the CNNEPDNN model were no less than those of the CNN model except the inner race faults with fault sizes of 21 and 14 mils in Datasets C and D, and the ball faults with the fault size of 14 mils in Datasets B and D because the extracted features did not contain sufficient information for accurately distinguishing the same kind of the faults with different degrees of severity in rolling body and inner race. The signals of the same kind of faults with different degrees of severity were similar, thus it was more difficult to distinguish them than different kinds of faults.

#### 4.2.4. Feature Learning Ability

**Figure 11:**
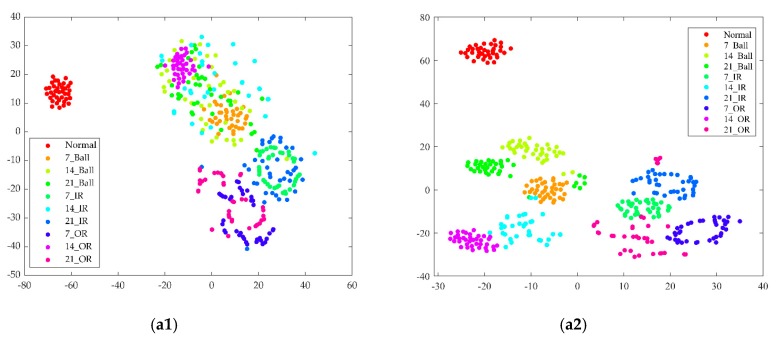

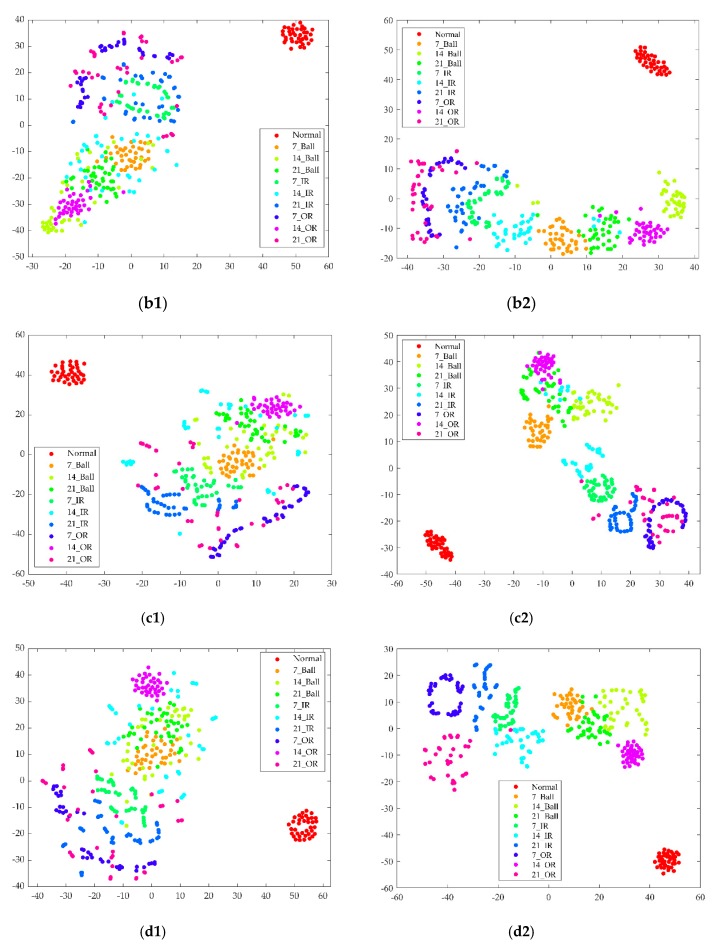
T-SNE visualization of features learned in the fully connected layer: (**a1**), (**b1**), (**c1**) and (**d1**) are the features of CNN learning from testing Dataset A, B, C and D, respectively. (**a2**), (**b2**), (**c2**) and (**d2**) are the features of CNNEPDNN learning from testing Dataset A, B, C and D, respectively.

The influences of fusion features obtained by t-SNE visualization on classification results are shown in Figure 11. In the 2D embedding figure, every point represents a sample and the axis represents the t-SNE dimension [35]. Figure 11(a1–d1) shows that the features learned from Datasets A–D at CNN corresponded to ten conditions. In addition to the features under normal conditions, the features of several other conditions overlapped with each other. The feature results of CNNEPDNN’s feature fusion layer learned in Dataset A, B, C and D are, respectively, shown in Figure 11(a2–d2). As shown in Figure 11(a2–d2), fusion features could be clustered well into categories and easily recognized, thus further confirming that the proposed model could improve the classification accuracy. However, as shown in Figure 11(b2–d2), the features of the ball faults with the depths of 7, 14 and 21 mils overlapped with the features of the fault of the inner race with a depth of 14 mils. The features of the outer race faults with a depth of 7 and 14 mils overlapped with the features of the inner race fault with a depth of 21 mils. The overlapping phenomena may be related to the extracted features and these types of faults could not be effectively identified.

### 4.3. Discussion

1. The experimental results show that the proposed model could effectively identify the same type of rolling bearing faults of different sizes. As shown in Figure 8 and Table 4, the proposed model and CNN have the best test accuracy and stability compared with DNN and BPNN under different motor loads. Experiments proved that CNN has the ability of automatic feature learning. The average test accuracies of CNNEPDNN model on Datasets A–D were, respectively, 3.04%, 0.51%, 0.13% and 2.36% higher than those of CNN model; and the standard deviations of CNNEPDNN model were, respectively, 0.34, 0.32, 0.19 and 0.45 lower than those of CNN model. We think that this result is significantly related to the structure of the proposed model. The proposed model integrated CNN and DNN in parallel. CNN extracts local features from the original vibration signal, DNN extracts waveform features from the time domain features, and further fuses these features to obtain the final result.

2. Although we integrate DNN in parallel on CNN, the training time of the proposed model was similar to CNN model. Through ten trials, we calculated the average training time of CNN was between 7 and 12 ms, and the average training time of DNN was 3 ms. The proposed model has a parallel structure and two local networks were trained at the same time, thus the average training time of the proposed model was similar to CNN. The loss function of the proposed model is cross-entropy, i.e., convex function. The model averages the output of the local model rather than the parameters, which guarantees the performance of the model. As shown in Figure 7, the proposed model converged more quickly than its local model.

3. The accuracy and reliability of the proposed model and CNN in fault identification of rolling bearings were further compared through F-score. In the confusion matrix shown in Figure 9, we can see the identification results of each type of fault. As shown in Table 5 and Figure 10, The F-score value of CNNEPDNN model was higher than the F-score value of CNN model. This proved that our model was effective. However, the inner race fault with fault size 21 and 14 mils in Datasets C and D, and the ball fault with fault size 14 mil in Datasets B and D were easily confused with each other. We think that this may be related to the insufficient feature extraction. The signals of the same kind of faults with different degrees of severity were similar, thus the features extracted from the local model did not distinguish the inner race fault and the ball fault.

4. Through T-SNE visualization, the feature learning abilities of the proposed model and CNN were further compared. As shown in Figure 11(a1–d2), by visualizing the features of the full-connection layer of CNN, the features of several other conditions overlapped with each other in addition to those under normal conditions. In the proposed model, the fusion features became distinguishable, as shown in Figure 11(a2–d2).

## 5. Conclusions and Future Work

In this study, we proposed a novel model CNNEPDNN to improve CNN in rolling bearing fault diagnosis. After integrating DNN with CNN, the extracted local features are fused with global features. The performance of the proposed fault diagnosis model for bearing fault was tested in ten conditions under different loads. The comparison of the diagnosis results of CNN and CNNEPDNN indicated that CNNEPDNN could give more precise diagnosis results for the same type of faults with different sizes. The visualized fusion features indicated that the feature clustering effect of the proposed model was better than that of CNN.

It is worth noting that the time domain features encounter some limitations, such as the bearings are running at variable speeds, or the noise is very high; it is difficult to design discriminant features or features become inconsistent, which will affect the diagnostic accuracy of DNN, and may affect the diagnostic accuracy of the whole model.

In the future, we will test the proposed model under more conditions. Furthermore, there are still some possible misclassifications. Additional features, sensor data, and other ensemble methods will be considered.

## Figures and Tables

**Table 1:** CNNEPDNN parameters.

Layers	CNN		DNN		Training Parameters
1	Input layer		Input layer	9	Adam Batch size = 100 Learning rate = 0.0015 Epoch = 100 (ks is kernel size; kn is kernel number; s is sub-sampling rate) Dropout = 0.5
2	Convolution layer 1	Ks = 5 × 1, Kn = 20, Stride = 1	Hidden layer 1	20	
3	Pooling layer	S = 2	Hidden layer 2	40	
4	Convolution layer 2	Ks = 5 × 1, Kn = 40, Stride = 1	Hidden layer 3	80	
5	Pooling layer	S = 2	Hidden layer 3	160	
6	Fusion layer	Relu activation function			
7	Softmax	10 outputs			

**Table 2:** Bearing dataset descriptions.

Fault Location		None	Inner Race			Outer Race			Ball		
Fault Diameter(mil)		0	7	14	21	7	14	21	7	14	21
Class label		0	1	2	3	4	5	6	7	8	9
Dataset A	Train	200	200	200	200	200	200	200	200	200	200
	Test	37	37	37	37	37	37	37	37	37	37
Dataset B	Train	200	200	200	200	200	200	200	200	200	200
	Test	37	37	37	37	37	37	37	37	37	37
Dataset C	Train	200	200	200	200	200	200	200	200	200	200
	Test	37	37	37	37	37	37	37	37	37	37
Dataset D	Train	200	200	200	200	200	200	200	200	200	200
	Test	37	37	37	37	37	37	37	37	37	37

**Table 3:** Features selected in the time domain.

Max	$x_{\max}=\max|x_{i}|$	Kurtosis	$q=\frac{1}{N}\sum_{i=1}^{N}{\left(x_{i}-\bar{x}\right)}^{4}$
Min	$x_{\min}=\min|x_{i}|$	Absolute mean	$x_{mean}=\frac{1}{N}\sum_{i=1}^{N}\left|x_{i}\right|$
Peak-Peak Value	$x_{F-F}=x_{\max}-x_{\min}$	Square root amplitude	$x_{t}={\left(\frac{1}{N}\sum_{i=1}^{N}\sqrt{\left|x_{i}\right|}\right)}^{2}$
Standard deviation	$\sigma =\sqrt{\frac{1}{N}\sum_{i=1}^{N}{\left(x_{i}-\bar{x}\right)}^{2}}$	Shape factor	$S_{f}=\frac{\sqrt{\frac{1}{N}\sum_{i=1}^{N}x_{i}^{2}}}{\frac{1}{N}\sum_{i=1}^{N}\left|x_{i}\right|}$
Skewness	$g=\frac{1}{N}\sum_{i=1}^{N}{\left(x_{i}-\bar{x}\right)}^{3}$		

Note: N is the number of sampling points and $x_{i}$ is the amplitude of the signal at each sampling point.

**Table 4:** Average testing accuracy and standard deviation of comparative methods.

Dataset	CNNEPDNN		CNN		DNN		BPNN	
	Average Accuracy	Standard Deviation	Average Accuracy	Standard Deviation	Average Accuracy	Standard Deviation	Average Accuracy	Standard Deviation
A	98.10	0.94	95.07	1.28	89.89	1.63	80.43	1.36
B	97.62	0.42	97.11	0.74	89.46	1.32	83.07	1.43
C	97.92	0.44	97.79	0.63	86.32	1.98	82.41	1.06
D	95.76	0.70	93.40	1.15	83.07	1.43	79.40	1.40

**Table 5:** F-Score results obtained with CNN and CNNEPDNN.

Metric	Dataset A		Dataset B		Dataset C		Dataset D	
	CNNEPDNN	CNN	CNNEPDNN	CNN	CNNEPDNN	CNN	CNNEPDNN	CNN
Precision	0.99	0.97	0.98	0.98	0.99	0.98	0.99	0.97
Recall	0.99	0.97	0.98	0.95	0.99	0.98	0.99	0.97
F-Score	0.99	0.97	0.98	0.96	0.99	0.98	0.99	0.97
